# Enhancing Meaning in Life and Psychological Well-Being Among a European Cohort of Young Adults via a Gratitude Intervention

**DOI:** 10.3389/fpsyg.2021.751081

**Published:** 2022-01-04

**Authors:** Natalia Czyżowska, Ewa Gurba

**Affiliations:** ^1^Department of Pedagogy and Psychology, Institute of Psychology, Pedagogical University of Kraków, Kraków, Poland; ^2^Department of Philosophy, The Pontifical University of John Paul II, Kraków, Poland

**Keywords:** meaning in life, psychological well-being, young adults, gratitude, intervention, mental health

## Abstract

**Background:** Strengthening the sense of meaning in life and psychological well-being brings benefits for mental health. The group particularly vulnerable to mental problems are young adults, therefore the aim of our research was to explore how a gratitude intervention will affect the sense of meaning in life, psychological well-being, general health and perceived stress among them. The research also took into account the issue of expressing gratitude.

**Method:** The study involved 80 young adults (58 women and 22 men) who were randomly assigned to the experimental group that filled out the specially prepared diaries for a week (participants were asked to list three things for which they feel grateful, to whom they are grateful and if and how they expressed their gratitude) or the control group. Participants completed the Meaning in Life Questionnaire (MLQ), the General Health Questionnaire – 28 (GHQ-28), the Perceived Stress Scale (PSS), and the Ryff Scales of Psychological Well-Being (PWBS) twice (before and after intervention).

**Results:** In the experimental group significant increases were observed in three areas of psychological well-being: environmental mastery, relationships with others and purpose in life. The significant decrease was also noted in anxiety/insomnia and depression symptoms as well as in perceived stress. There were no differences in the level of meaning in life. There was a positive relationship between expressing gratitude and meaning in life and psychological well-being.

**Conclusion:** Proposed gratitude intervention has the potential to enhance psychological well-being among young adults, however, it may not be effective in enhancing meaning in life.

## Introduction

As research results have shown, prevention focusing solely on reducing the risk of mental disorders is insufficient and it is necessary to look for various ways to promote mental health (Keyes, 2007; Keyes et al., 2010) which is one of the goals of positive psychology. Positive psychology, in contrast to the general approach that pay a lot of attention to psychological disorders, maladaptive sides of human functioning and negative effects of stress, focuses on human virtues and positive traits. According to its pioneers, understanding and promoting the factors that allow individuals to thrive, is necessary to effectively prevent and treat psychopathology (Seligman and Csikszentmihalyi, 2000). This seems particularly important considering that mental health is more than the absence of symptoms of mental illness. According to the definition of the World Health Organization (2004), it is a state of well-being in which the individual knows his or her strengths and can cope with challenges of everyday life and contribute to the growth of his or her community. Mental health promotion should therefore include activities that could potentially enhance the sense of well-being. Increasing well-being is one of the basic goals of positive psychology interventions (PPIs; Carr et al., 2020), which do not completely replace traditional clinical psychological interventions, but complement them (Seligman and Csikszentmihalyi, 2000). In recent years there has been a significant increase of interest in such interventions (Weiss et al., 2016; Schotanus-Dijkstra et al., 2017; Koydemir et al., 2021; van Agteren et al., 2021). Young adults (between 18 and 29 years of age) appear to be one of the groups for whom such mental health promoting interventions should particularly be undertaken. As epidemiological data has shown the prevalence of any mental disorder among young adults was higher than in any other age group during the course of 12 months (Alonso et al., 2004; Ishikawa et al., 2018; Stagnaro et al., 2018; National Health Institute, 2019). This may be due to the fact that people in this age group are in a specific developmental period, transitioning between adolescence and adulthood, and the challenges associated with it may increase anxiety, insecurity and confusion (Arnett, 2014; Arnett et al., 2014).

In the psychological literature, there are two main approaches to well-being: *hedonistic* (which concentrates on subjective well-being understood as life satisfaction, associated with high levels of positive emotions and low levels of negative emotions) (Deci and Ryan, 2008) and *eudemonistic* (which concerns psychological well-being encompassing six dimensions of wellness which are related to optimal functioning: self-acceptance, positive relations with others, autonomy, environmental mastery, purpose in life and personal growth) (Ryff, 1995; Ryff and Keyes, 1995). One of the important elements of well-being is to perceive one’s own life as meaningful and valuable (Greenberg and Arndt, 2012). A sense of meaning in life is associated with better stress coping (Hooker et al., 2018), lower intensity of anxiety and depression symptoms (Disabato et al., 2017; Korkmaz and Güloğlu, 2021), and moreover, is regarded as a protective factor for mental health, e.g., reducing the severity of suicidal tendencies (Lew et al., 2020). Having meaning in life certainly brings many benefits to the functioning of the individual. However, as researchers point out, it is difficult to modify it directly, hence the idea to do so indirectly, e.g., by strengthening gratitude (Kleiman et al., 2013). It is known that both gratitude and meaning in life are positively related to psychological well-being (Krok, 2015; Kardas et al., 2019). Experiencing gratitude may contribute to living a meaningful life, as it is related, for example, to having a purpose in life and assessing one’s own life as more significant (Wood et al., 2009). Gratitude, understood in terms of life orientation, may also increase recognition for one’s own existence (Ryff and Singer, 1998). Research has also shown that meaning in life mediates the relationship between gratitude and well-being (Datu and Mateo, 2015). Furthermore, there is a negative relationship between gratitude and both depression and suicidal ideations (Liang et al., 2020; Lin, 2021) and those who are more grateful have greater life satisfaction (Xiang and Yuan, 2021). It is worth emphasizing that in the previous research on the relationship between gratitude, sense of meaning in life and well-being, the *experience* of gratitude was primarily measured. The researchers point out, however, that *expressing* gratitude, instead just experiencing it, may bring even more benefits to the individual (Lambert et al., 2010). Research results obtained so far show gratitude interventions increasing gratitude in groups of young adults (Baumsteiger et al., 2019; Koay et al., 2020). There is also a single study that shows that gratitude interventions can strengthen purpose in life, both in terms of its search and identification, in this age group (Bronk et al., 2019), which allows to assume that they may also have the potential to strengthen the sense of meaning in life. However, there is no data that would indicate that.

The first aim of our research was to examine whether a gratitude intervention would enhance the sense of meaning in life and psychological well-being as well as contribute to the reduction of undesirable symptoms such as anxiety and depression. It was decided to focus on the group of young adults (between 18 and 29 years of age) as the group particularly vulnerable to mental problems. The second aim of this study was to investigate the relationship between expressing gratitude, meaning in life, general health, perceived stress and psychological well-being.

## Method

### Procedure

The research was designed as a pretest – posttest control group study. Each participant met the researcher twice. During the first meeting, participants drew an envelope with a code assigning them to one of two groups – with or without intervention (control group). The envelopes with the codes were arranged in a random order. Participants from intervention group received specially prepared paper diaries in which they were to write down every day for 7 days three things for which they feel grateful, to whom they are grateful and if and how they expressed their gratitude in these situations. In order to not influence the answers given in the questionnaires, the respondents were not informed that the intervention might affect their sense of meaning in life or well-being before the end of the study. Participants from control group did not perform any additional activities during the week. A week later, a second meeting with the researcher took place. One could receive a salary of $9 for participating in the study.

The study was conducted in accordance with the principles of the Declaration of Helsinki. Participants received oral and written information about the study and signed informed consent to participate in the study. The study was voluntary and anonymous, participants could withdraw at any time without giving any reason. The project was approved and financed from the funds earmarked for young scientists and doctoral students at the Faculty of Philosophy of the Jagiellonian University in Kraków.

### Participants

Eighty young adults (56 women and 22 men) between 18 and 25 years old participated in the study. The respondents were recruited via e-mail and through advertisements posted on student forums of three universities in Kraków. The study involved people who were not undergoing psychiatric treatment. People who experienced a traumatic event in the previous year (such as a divorce, accident, death of a loved one) were excluded from the study due to the fact that it could significantly affect their sense of meaning in life. The research was conducted from January to October 2019. The results of *a priori* analysis of statistical power for differences between dependent means (matched pairs) with effect size defined as *q* = 0.5 showed that for error probability set as α = 0.05 and power set as 1-β = 0.9 the minimum required sample size was 36. The results of *a priori* analysis of statistical power calculated for linear multiple regression with effect size defined as *f*^2^ = 0.5 showed that for error probability set as α = 0.05, power set as 1-β = 0.9 and number of predictors set as 3 the minimum required sample size was 33. Detailed information on the experimental group and the control group are presented in Table 1.

**TABLE 1:** Characteristics of the sample (*n* = 80).

	Group with intervention		Control group	
	*M*	SD	*M*	SD
Age	20.60	1.83	21.25	2.29

	***n***	**%**	***n***	**%**

**Sex**				
Male	7	17.5	15	37.5
Female	33	82.5	23	62.5
**Place of residence**				
Town	27	67	35	87.5
Village	13	33	5	12.5
**Marital status**				
Single	26	65	17	43
Informal relationship	14	35	23	57
Married	0	0	0	0
**Children**				
Yes	0	0	0	0
No	40	100	40	100
**Job**				
Yes	11	27.5	14	36
No	29	72.5	26	64

### Measures

The respondents completed all the questionnaires twice (except for the short demographic questionnaire which included questions about gender, marital and parental status, place of residence, employment status): before the start of the intervention and after 1 week (i.e., at the end of the intervention). All questionnaires are commonly used in scientific research and have sufficient psychometric values.

#### Meaning in Life Questionnaire

Meaning in Life Questionnaire by Steger et al. (2006) contains 10 questions rated on a 7-point Likert scale (from “absolutely untrue” to “absolutely true”). It consists of two subscales: presence of meaning in life and search of meaning in life, which allow to measure the sense of meaning in life in the present and in the future. The research used the Polish version of the questionnaire. Cronbach’s alpha index for the subscale measuring the presence of meaning in life is 0.86 and for the subscale used to measure sense-seeking is 0.87 (Kossakowska et al., 2013).

#### General Health Questionnaire-28

General Health Questionnaire-28 by Goldberg and Hillier (1979) consists of four subscales (7 items each) allowing the measurement of: the severity of somatic symptoms (subscale A), anxiety and insomnia (subscale B), social dysfunction (subscale C), symptoms of depression (subscale D). The severity of symptoms is rated by the subject on a 4-point scale (from “not at all” to “much more than usual”). Polish adaptation of the questionnaire was used in the study where Cronbach’s alpha index for subscales ranges from 0.82 to 0.93 (Makowska and Merecz, 2001) Perceived Stress Scale (PSS).

Perceived Stress Scale by Cohen et al. (1983) is used to measure feelings and reactions related to everyday problems and ways of coping. It consists of 10 questions rated on a scale from 0 – “never” to 4 – “very often.” The study used the Polish adaptation of the questionnaire where Cronbach’s alpha index was 0.86 and test-retest reliability (4-week period) equaled 0.72 (Juczyński and Ogińska-Bulik, 2009).

#### Ryff’s Psychological Well-Being Scales

Psychological Well-Being Scales by Ryff and Keyes (1995) are used to measure six dimensions of psychological well-being in the eudemonistic approach: autonomy, self-acceptance, positive relationships with others, personal development, life goal and environmental mastery. The scales consist of 84 items rated from 1 to 6 (1-“I strongly disagree”; 3-“I rather disagree,” 6-“I strongly agree”). As in the case of other tools, the Polish scale adaptation was used. For each subscale Cronbach’s alpha index is over 0.70 (Karaś and Cieciuch, 2017).

#### Expressing Gratitude Index

Inquiring whether the participants expressed gratitude allowed us to calculate the rate of expressing gratitude. For each situation in which the respondent expressed gratitude in some way, 1 point was awarded – thus, the maximum was 21 points (three daily situations in which the respondent could express gratitude multiplied by 7 days of the intervention).

### Statistical Analyses

The distribution of all variables (except for depression symptoms) was approximately normal as skewness and kurtosis of the data were between −1 and +1 and *z* value was in the range of ±1.96 which is sufficient to establish normality of the data (Mishra et al., 2019). To compare the results obtained in the pre-test and post-test, paired samples Student’s *t*-tests were carried out. Due to the skewness of the distribution of depression symptoms, the Wilcoxon Test was used to compare the differences between first and second measurement. To investigate the relationship between expressing gratitude and other variables, the Pearson correlation coefficient was used. The error terms were normally distributed, as well as the other criteria of the regression analysis were met, which allowed to perform it (Flatt and Jacobs, 2019). All the statistical procedures were computed using STATISTICA 13.

## Results

In the experimental group, significant increases were noted between pretest and posttest in the following areas of psychological well-being: environmental mastery, positive relationships with others and purpose in life. The significant decrease was also observed on two GHQ-28 subscales: the B subscale (anxiety/insomnia) and the D subscale (depression symptoms) (*T* = 24.0; *z* = 2.85; *p* = 0.004) and in the area of perceived stress. There were no differences in the level of meaning in life. Detailed results are provided in Table 2.

**TABLE 2:** Comparison of the level of meaning in life, general health, perceived stress and psychological well-being before and after intervention in experimental group (with intervention) (*n* = 40).

		Before intervention		After intervention		*t*	*p*
		*M*	*S* *D*	*M*	*S* *D*		
Meaning in life (MLQ)	Total result	48.37	7.39	47.85	11.01	0.53	0.59
	Presence of meaning in life	21.15	5.02	21.75	6.64	–0.95	0.34
	Search of meaning in life	27.22	3.66	26.10	5.57	1.97	0.06
General Health Questionnaire (GHQ-28)	Total result	27.05	10.67	25.34	14.76	0.57	0.56
	Somatic symptoms	7.00	3.68	7.75	5.47	–1.02	0.31
	Anxiety and insomnia	8.00*	3.57	7.20*	4.30	2.22*	0.03*
	Social dysfunction	7.32	3.36	7.45	2.85	–0.50	0.61
	Depression symptoms	3.97	5.02	3.17	5.11	–	–
Perceived stress (PSS)	Total result	20.65*	6.67	19.00*	6.57	2.27*	0.02*
Psychological well-being (PWBS)	Total result	57.57	9.48	57.33	9.15	0.04	0.96
	Self-acceptance	50.97	14.86	51.75	16.43	–0.89	0.37
	Positive relationships with others	57.25***	9.83	62.05***	10.31	−6.14***	< 0.001***
	Autonomy	56.45	11.25	56.57	13.24	–0.13	0.89
	Environmental mastery	53.57**	10.12	55.32**	10.29	−2.63**	0.01**
	Purpose in life	54.00***	8.47	57.82***	10.58	–4.52	< 0.001***
	Personal growth	63.92	6.99	63.97	8.22	–0.09	0.92

*Statistically significant results; *p ≤ 0.05; **p ≤ 0.01; ***p ≤ 0.001.

In the control group, the level of perceived stress increased after a week, as well as the results on GHQ-28 subscales: the B subscale (anxiety/insomnia) and the C subscale (social dysfunction). There were no differences in the level of meaning in life and psychological well-being. Detailed results are provided in Table 3.

**TABLE 3:** Comparison of the level of meaning in life, general health, perceived stress and psychological well-being before (pretest) and after a week (posttest) in control group (without intervention) (*n* = 40).

		Pretest		Posttest		*t*	*p*
		*M*	*S* *D*	*M*	*S* *D*		
Meaning in life (MLQ)	Total result	47.50	5.56	47.75	6.03	–1.74	0.10
	Presence of meaning in life	22.12	3.98	22.25	3.95	–0.11	0.91
	Search of meaning in life	25.37	4.52	26.50	3.89	–1.88	0.07
General Health Questionnaire (GHQ-28)	Total result	23.33	9.79	25.00	7.48	–0.39	0.70
	Somatic symptoms	8.00	3.50	6.75	2.35	1.53	0.14
	Anxiety and insomnia	7.75	3.37	8.75	3.82	–1.41	0.17
	Social dysfunction	6.62*	1.45	7.50*	0.73	−2.67**	0.01**
	Depression symptoms	1.25	1.23	0.87	1.20	–	–
Perceived stress (PSS)	Total result	16.25*	5.02	18.25*	6.27	−2.48*	0.02*
Psychological well-being (PWBS)	Total result	60.31	3.64	60.64	4.62	–0.64	0.52
	Self-acceptance	61.37	4.03	62.38	5.48	–0.67	0.50
	Positive relationships with others	59.12	9.05	58.75	4.84	0.28	0.78
	Autonomy	58.87	13.24	57.87	12.37	0.99	0.33
	Environmental mastery	60.00	5.77	59.37	4.86	0.67	0.50
	Purpose in life	57.25	5.90	59.62	7.62	–3.88	0.08
	Personal growth	65.25	4.72	62.37	7.29	1.58	0.13

*Statistically significant results; *p ≤ 0.05; **p ≤ 0.01.

There was a positive relationship between expressing gratitude and meaning in life and psychological well-being, and a negative relationship between anxiety/insomnia, social dysfunction and depression symptoms. Detailed results are provided in Table 4.

**TABLE 4:** Spearman R correlations among expressing gratitude index and meaning in life, general health, perceived stress, psychological well-being (*n* = 40).

		Expressing gratitude index
Meaning in life (MLQ)	Total result	0.43*
	Presence of meaning in life	0.54**
	Search of meaning in life	0.12
General Health Questionnaire (GHQ-28)	Somatic symptoms	–0.05
	Anxiety and insomnia	−0.44*
	Social dysfunction	−0.39*
	Depression symptoms	−0.45*
Perceived stress (PSS)	Total result	–0.29
Psychological well-being (PWBS)	Total result	0.39*
	Self-acceptance	0.46**
	Positive relationships with others	0.21
	Autonomy	0.29
	Environmental mastery	0.41*
	Purpose in life	0.41*
	Personal growth	0.36

*Statistically significant results; *p ≤ 0.05; **p ≤ 0.01.

The overall model *F* test for the multiple regression conducted to predict the global psychological well-being from meaning in life, perceived stress and general health was significant *F*_(3_,_76)_ = 8.45, *p* = 0001, *R*^2^ = 0.327, *R*^2^_adj_ = 0.29, however, only meaning in life (β = 0.35; *p* = 0.005) and perceived stress (β = −0.33; *p* = 0.007) were statistically significant predictors.

## Discussion

The findings demonstrated that the gratitude intervention improved psychological well-being, specifically in the areas of environmental mastery, positive relationships with others and purpose in life among a sample of young adults studying in Europe. This is consistent with the results obtained by other researchers, which indicate that gratitude interventions may increase well-being in this age group. However, it is worth emphasizing that most of these studies concerned life satisfaction, not the six dimensions of psychological well-being (Lyubomirsky et al., 2011; Watkins et al., 2015; Gabana et al., 2019). The results of our study allow us to suppose that gratitude interventions may be useful both in the context of subjective and psychological well-being. A decrease in symptoms of anxiety/insomnia and depression was also observed in the intervention group. This is in line with other studies in which the effectiveness of gratitude interventions for anxiety and depression symptoms was tested (O’Leary and Dockray, 2015; Heckendorf et al., 2019). However, it is noted that the effectiveness of gratitude interventions in reducing such symptoms is limited and rather low (Cregg and Cheavens, 2021). In our research we also noted a decrease in perceived stress in the intervention group. The research results in this area are not consistent – there are reports indicating that gratitude interventions are effective in reducing perceived stress (Killen and Macaskill, 2015; O’Leary and Dockray, 2015) and those that do not record significant changes (Koay et al., 2020). There were no significant differences in the level of meaning in life, so our gratitude intervention has proved ineffective in this area. There is much evidence that there is a positive relationship between gratitude and meaning in life (Kleiman et al., 2013; Datu and Mateo, 2015; Disabato et al., 2017), but the effectiveness of gratitude interventions for meaning in life has not yet been studied. Researchers point out that meaning in life is rather stable (Steger and Kashdan, 2006), which may make it not so easy to strengthen with simple interventions. Researchers suggest that the process of creating/maintaining meaning is complex and dynamic and its strengthening should be associated with increasing one’s self-awareness and the possibility of a different view of oneself and one’s own life. For that reason autobiographical methods could be more useful and adequate (Reker et al., 2013). It is worth emphasizing, however, that our intervention was short, what could have contributed to the lack of the expected results. Our research also focused on expressing gratitude. There was a positive relationship between expressing gratitude and meaning in life and psychological well-being, and a negative relationship between expressing gratitude and symptoms of anxiety and depression Taking into account the issue of expressing gratitude, encouraging it seems important because, as researchers emphasize, the beneficial effects of gratitude can only be fully realized when it is expressed outwardly (Lambert et al., 2010). As expressing gratitude is considered one of the most potent ways to practice it (Lambert et al., 2013) people who do not share their gratitude with benefactors may not derive optimal benefits from gratitude interventions (Davis et al., 2016). Thus, it seems a good idea to implement interventions where participants would be encouraged to express their gratitude in various ways. The conducted analyses showed that meaning in life and perceived stress are predictors of psychological well-being. There are single reports indicating that meaning in life is a predictor of psychological well-being, although they used a different tool to measure meaning in life (García-Alandete, 2015). Studies with Iranian female adolescents showed that perceived stress is one of the predictors of psychological well-being, although it should be emphasized that it was a very specific group (Hezomi and Nadrian, 2018), so further research is needed in this area.

The study has some limitations. Firstly, the studied sample was relatively small and related to a specific developmental period, which means that the observed relationships do not necessarily apply to other age groups. The study group was also homogeneous (young, childless students) which could have influenced the obtained results. In future studies it would be worthy to verify the effectiveness of interventions among people in other life situations. Secondly, women predominated among the study participants and gender may be one of the factors moderating the effectiveness of interventions and the strength of the described relationships, so future research should ensure an equal distribution of gender among the respondents. The proposed intervention was relatively short, so extending its duration, e.g., to 14 or 21 days may be worthwhile in future studies examining its effectiveness. Moreover, follow-up study was not conducted, so it cannot be determined whether there are long-term effects of this intervention and whether the desired changes persist for a long time, which is very important in the context of preventive and therapeutic interventions.

Gratitude interventions seems to be a promising way of enhancing the sense of psychological well-being and reducing the symptoms of anxiety and depression among young adults, especially that it is very easy to implement in everyday life, it does not take much time and does not require financial outlays, although more research is needed in this area. Taking into account the positive relationships between expressing gratitude and meaning in life and psychological well-being, it also seems that planned interventions should encourage people not only to experience gratitude, but also to express it.

## Data Availability Statement

The raw data supporting the conclusions of this article will be made available by the authors, without undue reservation.

## Ethics Statement

Ethical review and approval was not required for the study on human participants in accordance with the local legislation and institutional requirements. The patients/participants provided their written informed consent to participate in this study.

## Author Contributions

NC and EG: conceptualization and writing – review and editing. NC: data curation, formal analysis, methodology, investigation, visualization, and writing – original draft. EG: supervision. Both authors contributed to the article and approved the submitted version.

## Conflict of Interest

The authors declare that the research was conducted in the absence of any commercial or financial relationships that could be construed as a potential conflict of interest.

## Publisher’s Note

All claims expressed in this article are solely those of the authors and do not necessarily represent those of their affiliated organizations, or those of the publisher, the editors and the reviewers. Any product that may be evaluated in this article, or claim that may be made by its manufacturer, is not guaranteed or endorsed by the publisher.
